# Supplementary material for: Role of SiPHR1 in the Response to Low Phosphate in Foxtail Millet via Comparative Transcriptomic and Co-Expression Network Analyses
Source: Int J Mol Sci. 2023 Aug 14;24(16):12786. doi: 10.3390/ijms241612786 (PMC10454940; doi:10.3390/ijms241612786)
Supplement: Supplementary file 1 [file ijms-24-12786-s001.zip › ijms-2469279-supplementary.pdf]

Supplementary Material

Table S1. Gene specific primers used in this study.

Gene ID	Primer sequence	Products（bp）
Si0g10340	F: GCAGAGGTAGCAAGGAAG<br>R: ATCCAGGCACTATCCATCT	160
Si1g04550	F: GCTGATGTTCTCGTCCAA<br>R: GATTGCTTGTCCTCGTTG	132
Si1g33010	F: CCAGCCATTACCATGAAGT<br>R: AAGTTGCCGTACCAGATG	80
Si2g29010	F: ATGGCTACGGCAATTACAA<br>R: TGGCAGTGGAATATGGAAG	260
Si2g30500	F: GAGCATACCGTGGACTTC<br>R: TACCAACAGCACCAGAGA	118
Si2g42340	F: TCCGATTGTTGTGCTCTG<br>R: CCATCTCTTCCTGTTGTCTT	135
Si1g27930	F: TCCTGTGCTAGTGTATGATG<br>R: GCTGGACCTTGTTCTCAA	101
Si3g15010	F: TGAAGAAGGTCCAACAAGAA<br>R: AGAAGGCTGAGGCATACT	265
Si9g40880	F: ACCAAGAGCACCTGTCAT<br>R: AATCAGTCTCCGCATCTAAG	160
Si1g33510	F: CAAGTTCGGCTTCAACCT<br>R: GTTCGGCATCCTCTTCTC	238
Si9g08330	F: AGTCCTTCAGCTCCTACC<br>R: AACTCCTTGTGGCAGATG	244
Si9g36610	F: GGAGGAGGAGTTCGTCAT<br>R: TAGTTGATGCTGCTGTAGTT	168
Si4g21330	F: ATACATCATCCGCCAGAAG<br>R: AATCTTAACCAGTCCAGTGT	166
Si1g05330	F: CAAGAGCCTGAACAACCA<br>R: CGTAGTCCTCCTCCTTCT	254
Si9g03550	F: ATGCGAGGCTAATCTTGAA<br>R: GAGTCTGTTGAGGAATCTGA	300
****EF-Iα***	F: CAACAAGATGGATGCCACCAC<br>R: GAGATTGGGACGAAGGCAATC	126

Note: F indicated forward primer, R indicated reversed primer. * Indicated reference genes for qRT-PCR.

Table S2. Quality evaluation of RNA sequencing data.

Samples	Obtained Reads	Obtained Base(bp)	Q20(%)	Q30(%)	GC (%)
2h-NP-shoot-1	22,044,237	6,604,649,926	97.45	93.33	53.56
2h-NP-shoot-2	22,064,858	6,610,969,316	97.47	93.36	53.77
2h-NP-shoot-3	29,444,473	8,817,752,674	98.12	94.68	53.95
2h-LP-shoot-1	21,351,204	6,385,763,052	98.16	95.03	54.1
2h-LP-shoot-2	23,300,811	6,961,457,994	98.29	95.29	53.97
2h-LP-shoot-3	29,608,416	8,843,738,228	98.13	94.95	53.91
24h-NP-shoot-1	23,304,175	6,967,351,440	98.14	94.69	53.46
24h-NP-shoot-2	20,741,737	6,215,794,212	97.4	93.16	53.48
24h-NP-shoot-3	22,156,848	6,634,540,696	97.43	93.26	53.35
24h-LP-shoot-1	21,809,628	6,522,741,762	98.12	94.9	52.95
24h-LP-shoot-2	23,321,012	6,975,430,022	98.11	94.89	54.17
24h-LP-shoot-3	25,913,699	7,746,854,100	98.11	94.86	53.19
72h-NP-shoot-1	21,232,572	6,348,117,758	98.15	95.03	53.26
72h-NP-shoot-2	23,219,142	6,943,030,854	98.19	95.08	53.23
72h-NP-shoot-3	25,197,404	7,528,168,316	98.13	94.96	53.36

72h-LP-shoot-1	20,072,632	5,995,424,694	98.15	94.96	53.18
72h-LP-shoot-2	23,944,395	7,157,410,432	98.08	94.82	53.18
72h-LP-shoot-3	22,754,731	6,798,538,878	98.12	94.87	53.02
2h-NP-root-1	22,923,071	6,857,602,698	97.9	94.45	54.04
2h-NP-root-2	22,878,889	6,855,453,236	97.43	93.48	53.68
2h-NP-root-3	21,395,888	6,410,865,730	97.4	93.51	53.77
2h-LP-root-1	21,684,136	6,485,889,104	97.61	94.13	53.41
2h-LP-root-2	23,700,573	7,089,350,452	97.8	94.4	53.25
2h-LP-root-3	22,805,084	6,824,576,230	97.77	94.37	53.28
24h-NP-root-1	22,939,028	6,871,756,610	97.14	92.89	52.63
24h-NP-root-2	23,232,348	6,947,990,350	97.71	94.22	52.42
24h-NP-root-3	23,428,644	7,004,518,664	97.86	94.56	53.28
24h-LP-root-1	23,221,563	6,949,916,072	97.66	93.84	53.43
24h-LP-root-2	22,895,798	6,864,428,138	97.46	93.55	53.67
24h-LP-root-3	21,030,182	6,287,232,190	97.89	94.33	53
72h-NP-root-1	22,646,358	6,772,069,754	97.78	94.36	53.55
72h-NP-root-2	23,557,700	7,044,169,624	97.92	94.68	53.47
72h-NP-root-3	24,145,864	7,214,852,034	97.92	94.44	54.13
